# Poly(Ionic Liquid)-Based Composite Electrolyte Membranes: Additive Effect of Silica Nanofibers on Their Properties

**DOI:** 10.3390/membranes15090254

**Published:** 2025-08-27

**Authors:** Yoshiki Kawai, Yirui Lu, Shaoling Zhang, Gen Masuda, Hidetoshi Matsumoto

**Affiliations:** 1 Department of Materials Science and Engineering, School of Materials and Chemical Technology, Institute of Science Tokyo, 2-12-1 Ookayama, Meguro-ku, Tokyo 152-8552, Japan; 2 School of Materials Science and Engineering, Tsinghua University, Beijing 100084, China; 3 Nisshinbo Holdings Inc., 1-2-3 Onodai, Midori-ku, Chiba-shi 267-0056, Japan

**Keywords:** poly(ionic liquid), silica nanofiber, imidazolium hydrogen sulfate, polymer electrolyte membrane, proton conductivity, mechanical properties, thermal property

## Abstract

Poly(ionic liquids) (PILs) show great promise as a new class of solid electrolytes for energy applications, including high-temperature polymer electrolyte fuel cells, owing to their combination of the unique electrochemical properties of ionic liquids and macromolecular architecture. In this study, we prepared and characterized PIL-based composite polymer electrolyte membranes containing silica nanofibers (SiO_2_NFs). The SiO_2_NFs were prepared via electrospinning, followed by calcination, and were used as a thermally and mechanically stable, porous substrate. The crosslinked protic PIL was synthesized via in situ radical polymerization of imidazolium hydrogensulfate-based reagents (one monomer and one crosslinker). It was then used as the membrane matrix. The prepared freestanding PIL membranes remained thermally stable at temperatures of up to 180 °C. Furthermore, the PIL/SiO_2_NF composite electrolyte membranes demonstrated improved mechanical properties due to reinforcement by the NF framework. These composite membranes also exhibited relatively high proton conductivity (approximately 0.1 to 1 mS/cm) in the 100–150 °C temperature range.

## 1. Introduction

Polymer electrolyte fuel cells (PEFCs) are attracting increasing attention due to their high energy conversion efficiency, power density, and zero CO_2_ emissions. Remarkable progress has been made in FC technology over the past few decades. PEFCs are currently being commercialized for use in fuel cell vehicles and stationary power generation systems [1,2,3]. However, further commercialization still faces several challenges. One major issue is performance degradation during operation, which is caused by various complex factors, such as the degradation of electrode materials, a loss of catalysts, and mechanical damage to the membrane electrode assembly [4]. Another important issue is system cost. Recently, operating PEFCs at temperatures above 100 °C without humidification has been expected as a means of reducing system costs. Such high-temperature operation offers several practical advantages, such as improved CO_2_ tolerance, simplified water and heat management, and increased coverage of catalyst choices, including non-platinum catalysts [5,6]. However, conventional polymer electrolyte membranes (PEMs) based on perfluorosulfonic acid (PFSA) ionomers (e.g., Nafion) have poor proton conductivity above 100 °C. This is primarily due to membrane dehydration, which causes shrinking and disconnected ionic domains in the membranes under non-humidification conditions [7]. Therefore, the development of novel PEMs is essential for advancing high-temperature polymer electrolyte fuel cells (HT-PEFCs). Hydrocarbon-based PEMs are considered a promising low-cost, fluorine-free alternative to PFSA membranes. Many studies have focused on thermally stable hydrocarbon ionomers, such as sulfonated polyphenylenes and phosphoric acid-doped poly(benzimidazole) (PA-PBI), which perform well under low-humidity and high-temperature conditions [5,8,9]. A representative example is Celtec^®^, a commercially available PA-PBI membrane developed by BSAF [9].

Poly(ionic liquids) (PILs) are polyelectrolytes consisting of a polymeric backbone with ionic liquid (IL) moieties in the repeating units [10]. Many desirable properties of ionic liquids (ILs), such as negligible vapor pressure, nonflammability, thermal and chemical stability, wide electrochemical stability window, and high ionic conductivity [11], can be transferred from monomers to macromolecules. In addition, the designability of IL moieties and the combination of polymer segments further enhance the properties and applications of PILs. Therefore, PILs have received significant attention as solid electrolytes for energy applications, in particular for HT-PEFCs. For example, Ortiz et al. synthesized imidazolium-based PILs via photopolymerization of the IL monomer 1-(4-sulfonylbutyl)-3-vinylimidazolium trifluoromethanesulfonate, achieving thermal stability up to 200 °C and FC power generation under dry conditions [12]. Segalman et al. investigated a series of proton-conducting polystyrene-block-polymerized ionic liquids (imidazolium bis(trifluoromethane sulfonimide) copolymers spanning a range of molecular weights and compositions. They demonstrated that the primary factor governing high-temperature conductivity is the PIL content in the copolymer [13]. Zhu et al. developed a protic PIL membrane via the radical polymerization of the monomer [diethylmethylammonium][4-styrenesulfonyl(trifluoromethylsulfonyl) imide], which exhibited a conductivity of 0.3 mS/cm at 120 °C [14]. Wu et al. reported on a nanolayered aprotic PIL membrane prepared via in situ polymerization of imidazolium hydrogen sulfate liquid crystalline self-assemblies, achieving a proton conductivity of 22 mS/cm at 135 °C under anhydrous conditions [15]. More recently, Mecerreyes et al. successfully synthesized a protic PIL membrane based on poly(diallylmethylammonium) ([PDAMAH]^+^) with various anions, achieving an ionic conductivity value of 1.9 mS/cm at 120 °C under dry conditions [16]. In addition, Makhlooghiazad et al. proposed a PDAMAH membrane composed of a protic IL, which reduced its glass transition temperature and enhanced ionic conductivity [17]. These results clearly demonstrate the potential of PILs as PEMs for HT-PEFC applications. Our goal is to develop a protic PIL membrane that can be synthesized easily and maximizes ion density in the membrane. However, studies on easily synthesized crosslinked PILs, particularly crosslinked protic PILs, are still limited, probably due to their mechanical brittleness.

In this study, we prepared composite PEMs consisting of a crosslinked protic PIL matrix and thermally and mechanically stable silica nanofibers (SiO_2_NFs). The PIL matrix was synthesized via in situ radical polymerization of imidazolium hydrogensulfate-based reagents (one monomer and one crosslinker). The structure, thermal, mechanical, and electrochemical properties of the PEMs were then characterized. This work aims to investigate the additive effect of the SiO_2_NFs substrate on proton conductivity at high temperatures and mechanical properties.

## 2. Materials and Methods

### 2.1. Materials

1-Vinylimidazolium hydrogensulfate (monomer) and 1,4-(divinylimidazolium hydrogensulfonate) dodecane (crosslinker) were kindly provided by Nisshinbo Holdings Inc., Tokyo, Japan. These reagents were stored in an Ar-filled glovebox (<0.1 ppm of water and <0.1 ppm of O_2_). Poly(vinylpyrrolidone) (PVP, *M*_w_ = 1,300,000 g/mol) and potassium persulfate (initiator, purity ≥ 99.0%) were purchased from Sigma-Aldrich (St. Louis, MO, USA). Tetraethoxysilane (TEOS, purity ≥ 95.0%), 1 mol/L (M) of hydrochloric acid (HCl, for volumetric analysis), and 1-propanol (PrOH, purity ≥ 99.5%) were purchased from Fujifilm Wako (Osaka, Japan). Methanol (MeOH, purity ≥ 99.5%), ethanol (EtOH, purity ≥ 99.5%), sodium hydroxide (NaOH, purity ≥ 97.0%), acetone (Ac, purity ≥ 99.5%), acetonitrile (AN, purity ≥ 99.5%), and sulfuric acid (H_2_SO_4_, purity ≥ 96.0%) were purchased from Kanto Chemical (Tokyo, Japan). These reagents were used as received without further purification. Ultrapure water (H_2_O) was prepared by using a water purification system (Milli-Q Advantage, Merck Millipore, Burlington, MA, USA). The commercial PFSA membrane (Nafion^®^ NR-211) was purchased from Chemours (Wilmington, DE, USA). The membrane was used after boiling in H_2_O.

### 2.2. Preparation of Silica Nanofibers and Porous Substrate

Silica nanofibers (SiO_2_NFs) were prepared by electrospinning as previously reported [18,19]. TEOS, PVP (spinning aid), and PrOH were mixed and stirred at 25 ± 1 °C for 12 h. The HCl-H_2_O-PrOH solution was added to the mixture and stirred for 2 h to obtain a spinning solution. The final composition was TEOS:H_2_O:HCl:PrOH:PVP = 6.9:0.9:0.2:88.4:3.6 in weight. The spinning solution was then electrospun using a commercial device (ES-2000S, Fuence, Tokyo, Japan). A stainless-steel nozzle with an internal diameter of 0.31 mm, connected to a regulated direct current (DC) high-voltage power supply, was used as the spinneret. The grounded aluminum foil collector was used as the counter electrode. The applied voltage was 20 kV, the distance between the nozzle tip and the collector was 150 mm, and the flow rate was 3.8 µL/min. All spinnings were conducted at 25 ± 1 °C and under a relative humidity of less than 40%. The as-spun NF webs were calcined at 600 °C in a furnace for 6 h to remove the PVP and complete the condensation reaction to form SiO_2_.

The calcined SiO_2_NF webs were immersed in 0.4 M of NaOH for 2 min. After the neutralizing of the alkaline dispersion with 1M of HCl, the webs were vacuum-filtered on a poly(tetrafluoroethylene) (PTFE) membrane filter (0.2 μm pore size, Ominipore JGWPO04700, Merck Millipore, Burlington, MA, USA) and sufficiently washed with H_2_O, MeOH, and EtOH. Then they were vacuum-dried at 90 °C for 2 h. The treated SiO_2_NF webs were immersed in a mixture of AN and MeOH (2:1 in volume) and sonicated for 10 min using a low-power ultrasonic cleaner (40 kHz and 160 W, Branson CPX5800-J, Emerson, St. Louis, MO, USA) to separate the NF webs into the individual SiO_2_NFs. The obtained SiO_2_NF dispersion was then centrifuged at 4500 rpm for 20 min using a benchtop centrifuge (Sorvall ST8, Thermo Fisher Scientific, Waltham, MA, USA) to obtain the SiO_2_NF slurry by removing the supernatant. The SiO_2_NF slurry was cast on a glass plate with a PTFE spacer and dried under ambient conditions and then vacuum-dried at 100 °C for 2 h to obtain the porous SiO_2_NF substrate for membrane preparation.

### 2.3. Membrane Preparation

**Figure 1:**
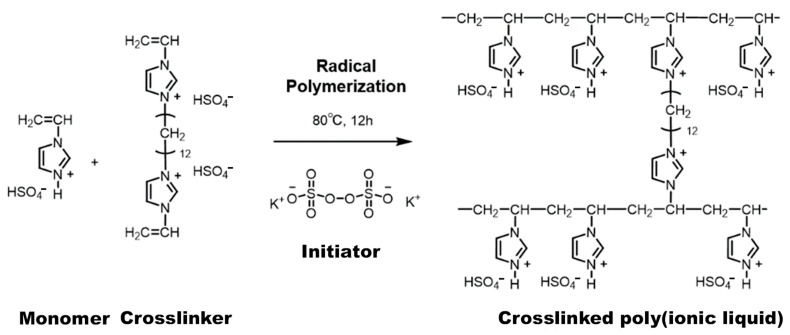
Schematic of synthesis of crosslinked protic poly(ionic liquid) by radical polymerization.

PIL was prepared by radical polymerization (see Figure 1). The monomer and crosslinker (the molar ratio of monomer to crosslinker is 93:7) were dissolved in H_2_O with/without H_2_SO_4_ (see Table 1 for the composition) and stirred at 300 rpm and 25 ± 1 °C. Then, the solution was filtered with cotton fabric (Bemcot M-1, Asahi Kasei, Tokyo, Japan). After adding initiator (0.6 mol% relative to the amount of vinyl groups in the solution) to the solution, the solution was cast onto the porous SiO_2_NF substrate and heated at 80 °C for 12 h to prepare PIL/SiO_2_NF composite membranes. For comparison, a pristine PIL membrane without containing SiO_2_NFs was similarly prepared by casting the solution on a glass substrate with a PTFE spacer and successive radical polymerization. All membrane preparations were carried out in an Ar-filled glovebox (<0.1 ppm of water and <0.1 ppm of O_2_). The prepared membranes were stored in the glovebox for over 12 h in order to ensure that the water in the membranes was eliminated. The membrane thickness was determined using a height gauge (ABSOLUTE T310111, Mitutoyo, Kawasaki, Japan).

### 2.4. Characterization

The morphologies of the prepared SiO_2_NFs, SiO_2_NF sheets, and membranes were observed by scanning electron microscopy (SEM, JCM-5700, JEOL, Akishima, Japan) operated at 5 kV and field-emission scanning electron microscopy (FE-SEM, JSM-7500F, JEOL, Akishima, Japan) operated at 3 kV. The insulating NF samples were sputter-coated with Pt, while the conductive membrane samples were observed without coating. The fiber diameter distribution was determined by SEM image analysis using ImageJ software (ver. 1.52a, National Institutes of Health, Bethesda, MD, USA). The pore size distribution, porosity, and total pore area of SiO_2_NF sheets were determined by mercury porosimetry measurements using an instrument (AutoPore 9520, Micrometrics, Norcross, GA, USA). The elemental compositions of the prepared membranes were analyzed via energy-dispersive spectroscopy (EDS) using an EDS spectrometer (AMETEK/EDAX Genesis, EDAX Inc., Pleasanton, CA, USA) attached to a field-emission scanning electron microscope (FE–SEM, SU9000, Hitachi High-Tech Corporation, Tokyo, Japan) operated at 6 kV. The samples were coated with osmium.

Wide-angle X-ray scattering (WAXS) measurements were performed at BL40B2 in the SPring-8 synchrotron radiation facility (Hyogo, Japan). The prepared membranes were irradiated with X-rays of wavelength (*λ*) = 0.1 nm. The scattering patterns were recorded on a PILATUS 3S 2 M detector (Dectris, Baden-Daettwil, Switzerland) located 114–324 mm from the sample.

The Fourier transform infrared (FTIR) spectra of the SiO_2_NFs and the prepared membranes were recorded by using an FTIR spectrometer (FT/IR-6300, JASCO, Hachioji, Japan) with an attenuated total reflection unit (PRO670H-S, JASCO, Hachioji, Japan), including a diamond crystal. Thermogravimetric analysis (TGA) curves of the prepared membranes were measured using a thermal analyzer (Thermo plus EVO TG 8120, Rigaku, Akishima, Japan) under an N_2_ atmosphere, by heating from 25 to 500 °C at a rate of 10 °C/min.

The through-plane ionic conductivity of the prepared membranes was measured using the alternating current impedance method with a potentio/galvanostat (SI 1287, Solartron, Farnborough, UK) in the range of 0.1 Hz to 1 MHz. The hand-made symmetrical two-electrode cells made of Pt (Figure S1) were used. The measurements were carried out at 150 to 100 °C under anhydrous conditions. To eliminate the influence of humidity, measurements were taken in the direction of cooling from the maximum temperature. The through-plane proton conductivity (*σ* [S/cm]) was calculated by the following equation:

(1) $$\sigma \;=\;\frac{L}{R\;\times \;A}$$

where *L* is the thickness of the membrane, *A* is the membrane area (area: 5 cm × 5 cm), and *R* is the membrane resistance.

The activation energy (*E*_a_) of the proton conductivity through the membranes was calculated using the following Arrhenius equation:

(2) $$\sigma \;=\;\sigma_{0}\exp(-\frac{E_{a}}{RT})$$

where *σ*_0_ is the value of the pre-exponential factor [S/cm], *E*_a_ is the activation energy required for protons to transport, *R* is the gas constant [J/(mol K)], and *T* is the absolute temperature [K].

Tensile tests of the prepared membranes were performed using a tensile tester (STA-1150, A&D, Tokyo, Japan). The test samples were cut to a 5 mm width and 15 mm length. All measurements were performed at an elongation rate of 1 mm/min at room temperature. Three samples were measured for each sheet, and the mean value (±standard error) was calculated.

## 3. Results and Discussion

### 3.1. Structures of SiO_2_NFs and SiO_2_NF Porous Substrates

**Figure 2:**
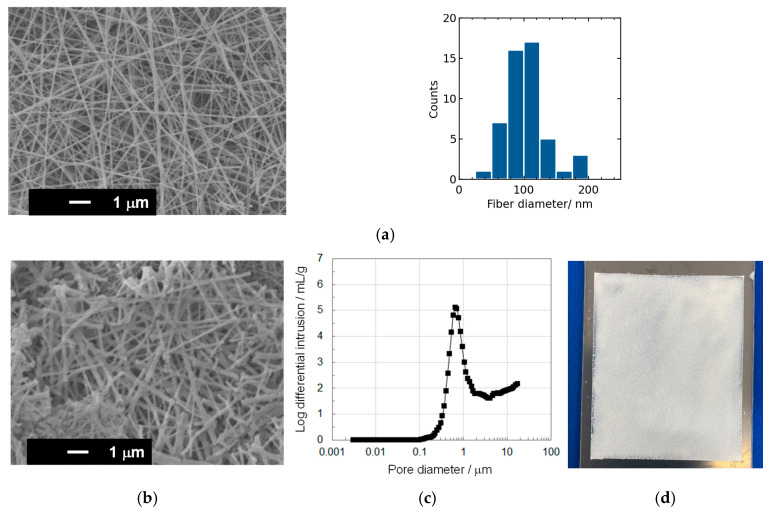
(**a**) Surface SEM image and fiber diameter distribution of the calcined SiO_2_NFs. (**b**) Surface SEM image, (**c**) pore size distribution curves, and (**d**) photograph of the SiO_2_NF porous substrate.

Figure 2a shows a surface SEM image and fiber diameter distribution for the calcined SiO_2_ NFs, revealing an average fiber diameter of 100 ± 30 nm. The FTIR spectrum is shown in Figure S2. The calcined SiO_2_NFs exhibit peaks at 1063 and 799 cm^−1^, which are attributed to the Si–O–Si asymmetric bond stretching vibration and the network Si–O–Si symmetric bond stretching vibration, respectively. The peaks at 3414 cm^−1^and 960 cm^−1^, which are assigned to the –OH stretching vibration and the Si–OH stretching mode typical of gel structure, respectively, were also observed [20]. Figure 2b,c show a surface SEM image and the pore size distribution of the SiO_2_NF sheet cast from SiO_2_NF slurry, respectively. The SiO_2_NFs form a dense and interconnected network in the sheet. The mean pore diameter, porosity, and total pore area were 0.62 µm, 89%, and 17 m^2^/g, respectively. The obtained SiO_2_NF sheet (Figure 2d) was used as the porous substrate for the preparation of PIL composite membranes.

### 3.2. Structures of Prepared PIL-Based Electrolyte Membranes

**Figure 3:**
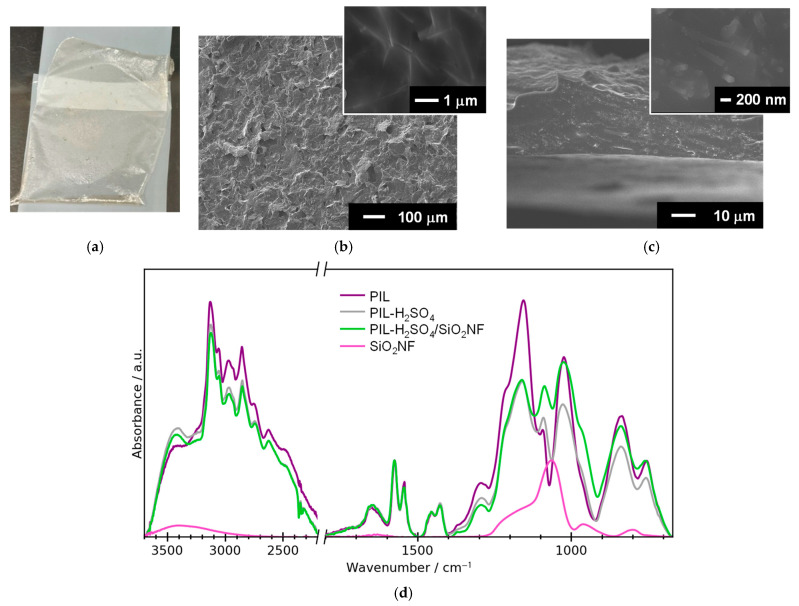
(**a**) Photograph, (**b**) surface, and (**c**) cross-sectional SEM images of the PIL-H_2_SO_4_ /SiO_2_NF composite membrane. The insets are high-magnification SEM images. (**d**) FT-IR spectra of the prepared membranes.

Figure 3 shows a typical photograph and SEM images of the PIL-H_2_SO_4_/SiO_2_NF composite membrane (the NF content of 10 wt%). The composite membrane exhibited high transparency (Figure 3a), had a rough surface (Figure 3b), and was approximately 50 μm thick (Figure 3c). The insets of Figure 3b,c clearly show white-colored SiO_2_NFs due to charging in the SEM. This finding supports the formation of a defect-free, dense microstructure with well-dispersed SiO_2_NFs in the polymer matrix. This microstructure is consistent with the transparency. Figures S3 and S4 show the EDS spectra and mapping of the surface and cross-section of the composite membrane. EDS analysis revealed the presence of the elements C, N, O, Si, and S in the composite membrane. EDS mapping clearly shows that the SiO_2_NFs are distributed throughout the membrane.

Figure 3d shows the FTIR spectra of the prepared membranes. Note that all peaks were normalized to the height of the in-plane vibration peaks of the imidazolium ring, observed at 1576 cm^−1^. All of the prepared membranes exhibited similar spectra. The observed IR peaks and their assignments are listed in Table S1 [21,22,23,24,25,26,27,28,29,30,31,32,33,34,35]. Figure S5 shows FTIR spectra of the monomer and PIL membranes. After polymerization, the characteristic peak at 1664 cm^−1^ from the C=C stretching vibration from the vinyl group of the monomer [25] decreased significantly due to the progress of the polymerization reaction. This confirms the success of the in situ polymerization.

**Figure 4:**
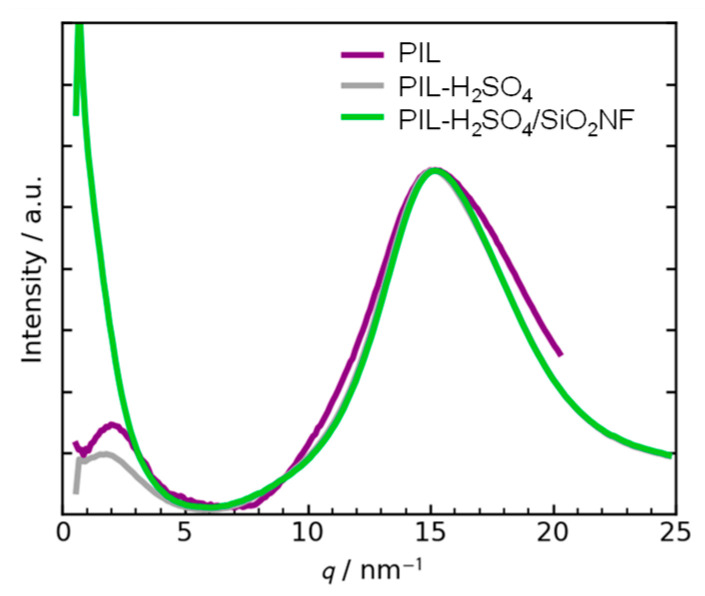
One-dimensional WAXS profiles of the prepared membranes.

Figure 4 shows the 1D WAXS profiles of the prepared membranes. A peak at 15 nm^−1^ (*d*-spacing of 0.42 nm) was observed for all membranes, which can be ascribed to the stacking of imidazolium rings [36]. A peak at approximately 2.1 nm^−1^ (*d*-spacing of 3.1 nm), corresponding to the distance between two neighboring macromolecular chains [37], was observed for the PIL membrane. The addition of H_2_SO_4_ weakened this peak, suggesting that adding an acid increases the distance between chains within the matrix. Additionally, it was found that SiO_2_NFs influence the formation of the microstructure of the matrix during membrane preparation [38].

**Figure 5:**
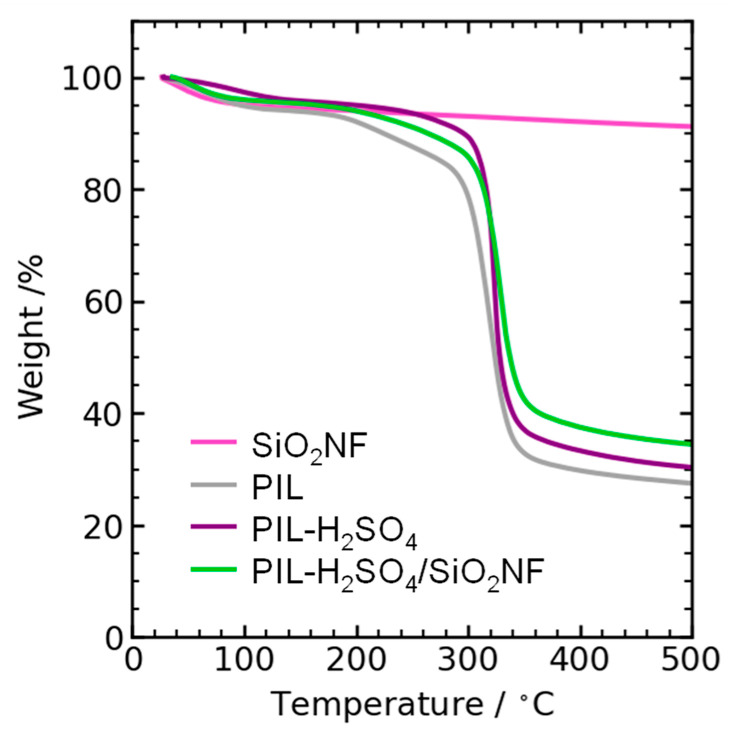
TGA curves of the prepared membranes.

Figure 5 shows the TGA curves of the prepared membranes. All membranes exhibited good thermal stability between 100 and 180 °C, and the weight loss up to 100 °C can be attributed to water evaporation. Therefore, we evaluated the proton conductivity through the membranes up to 150 °C.

### 3.3. Properties of the Prepared PIL-Based Electrolyte Membranes

**Figure 6:**
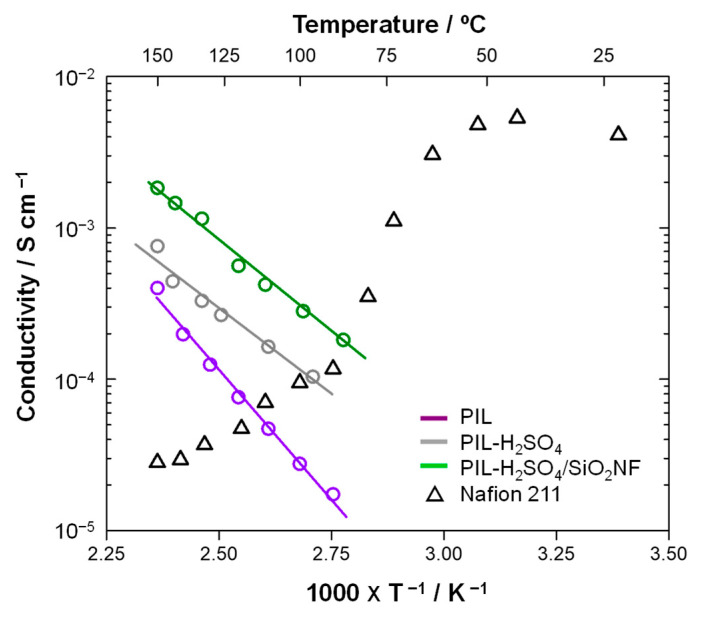
Temperature dependence of the proton conductivity through the prepared membranes. The symbol “circle” indicates experimental data.

Figure S6 shows typical Nyquist plots obtained from EIS measurements. Membrane resistance (*R*) was determined from the high-frequency intercept of the impedance arc on the real axis of the plots [39]. Figure 6 shows the temperature dependence of proton conductivity through the prepared membranes. All the prepared membranes showed increased conductivity above 80 °C. However, the conductivity of the Nafion membrane decreased in this region due to the disconnection of ionic domains. The order of proton conductivities through the membranes was as follows: PIL-H_2_SO_4_/SiO_2_NF > PIL-H_2_SO_4_ > PIL. Here, the activation energy (*E*_a_) for proton conductivity through the membrane was determined using Equation (2). The parameters, *σ*_0_ and *E*_a_, obtained by linear regression analysis of the plots in Figure 6 and Equation (2), are listed in Table S2. The *E*_a_ value for the PIL membranes was 65 kJ/mol. The value decreased to 44 and 47 kJ/mol for the PIL-H_2_SO_4_ and PIL-H_2_SO_4_/SiO_2_NF composite membranes, respectively. These decreases are attributed to the increased proton density within the membranes due to the addition of acid. As a result, the proton conductivity of the PIL-H_2_SO_4_-based membranes exceeded 0.1 mS/cm in the temperature range of 100–150 °C. Furthermore, the incorporation of SiO_2_NFs significantly enhanced proton conductivity, reaching 1.85 mS/cm at 150 °C. Notably, this improvement occurred despite the addition of 10 wt% NFs, which substantially reduced the apparent proton density in the membrane. No clear peak shifts indicating intermolecular interactions between the SiO_2_NFs and PIL matrix were observed in the IR spectra (see Figure 3). One possible explanation for the improved proton conduction is the microstructure change in the PIL matrix induced by the SiO_2_NFs (see Figure 4). However, these characterizations were performed at room temperature. Further detailed verification will be necessary, including small-angle and wide-angle X-ray scattering and FTIR measurements in the 100–150 °C temperature range.

**Figure 7:**
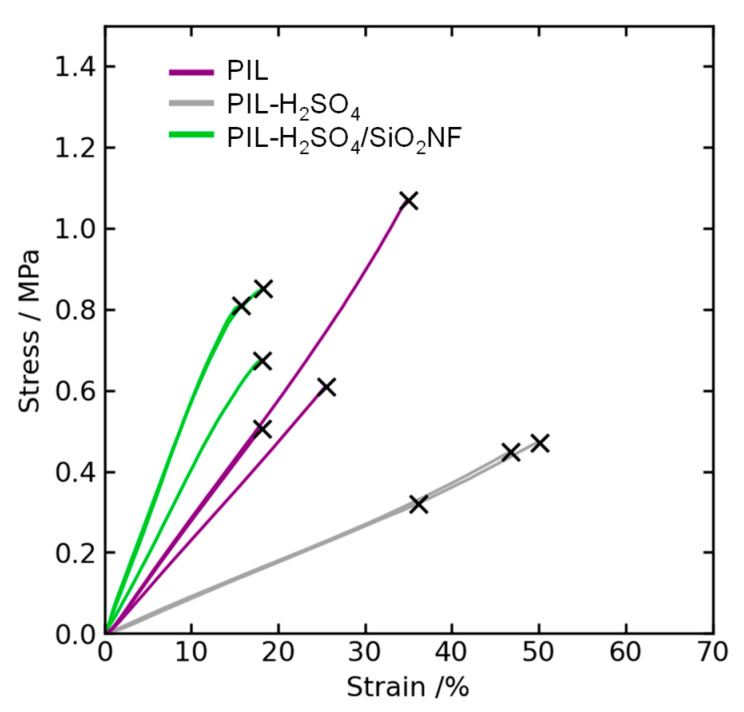
Stress–strain curves of the prepared membranes. The symbol “x” represents the breaking point of the samples.

The additive effects of the SiO_2_NFs on the mechanical properties of the prepared membranes (i.e., Young’s modulus, tensile strength, and elongation at break) were obtained from the stress–strain (S–S) curves (Figure 7). These results are summarized in Table 2. Compared to the PIL membrane, the PIL-H_2_SO_4_ membrane had a lower Young’s modulus and tensile strength, while the elongation increased. This is due to the lower crosslinker content of the PIL-H_2_SO_4_ membrane (see Table 1). Compared to the PIL-H_2_SO_4_ membrane, the Young’s modulus and tensile strength of the PIL-H_2_SO_4_/SiO_2_NF composite membrane increased by 5.7 and 1.9 times, respectively. Membrane elongation decreased from 44% to 17% with the addition of NFs. These results clearly indicate that the rigid SiO_2_NF framework reinforces the crosslinked PIL matrix and restricts its deformation.

## 4. Conclusions

In this work, crosslinked protic PIL-based electrolyte membranes were prepared via in situ radical polymerization of imidazolium hydrogensulfate-based reagents (one monomer and one crosslinker). The prepared freestanding PIL membranes exhibited thermal stability up to 180 °C and showed increasing anhydrous proton conductivity at temperatures above 90 °C. Adding sulfuric acid improved proton conductivity but decreased mechanical strength. The addition of SiO_2_NFs improved both proton conductivity and mechanical strength. The PIL-H_2_SO_4_/SiO_2_NF composite membrane exhibited a maximum proton conductivity of 1.85 mS/cm at 150 °C. These results clearly indicate the potential of composite PEMs consisting of a crosslinked protic PIL matrix and a thermally and mechanically stable NF substrate. However, the mechanical strength of the PIL-H_2_SO_4_/SiO_2_NF composite membrane is still insufficient from the viewpoint of actual fuel cell applications. Further improvements in mechanical properties are expected through the rational tuning of structural parameters, such as increasing the volume fraction of SiO_2_NFs and/or incorporating other thermally and mechanically stable NF materials. Additionally, improving proton conductivity requires an in-depth understanding of the additive effect of SiO_2_NFs on the crosslinked PIL matrix. Further studies on the precise characterization of the composite membrane in the 100–150 °C temperature range are currently in progress, and the results will be reported.

## Figures and Tables

**Table 1:** Composition of the monomer solution for radical polymerization.

Membrane	Monomer [wt%]	Crosslinker [wt%]	H_2_O [wt%]	H_2_SO_4_ [wt%]	Initiator [wt%]
PIL	41.65	8.35	41.65	-	0.42
PIL-H_2_SO_4_	41.65	8.35	41.65	8.35	0.42
PIL-H_2_SO_4_/SiO_2_NF	41.65	8.35	41.65	8.35	0.42

Here, the components of the PIL-H_2_SO_4_ membrane, excluding the initiator, were set to 100%.

**Table 2:** Mechanical properties of the prepared membranes.

Membrane	Young’s Modulus [MPa]	Tensile Strength [MPa]	Elongation at Break [%]
PIL	2.8 ± 0.2	0.73 ± 0.20	26 ± 5
PIL-H_2_SO_4_	0.90 ± 0.01	0.41 ± 0.05	44 ± 4
PIL-H_2_SO_4_/SiO_2_NF	5.1 ± 0.6	0.78 ± 0.05	17 ± 1

Measured under ambient conditions (@20 °C and 40%RH).

## Data Availability

The original contributions presented in this study are included in the article and Supplementary Material. Further inquiries can be directed to the corresponding author.

## Supplementary Materials

The following supporting information can be downloaded at https://www.mdpi.com/article/10.3390/membranes15090254/s1, Figure S1: Schematic of the measuring cell for high-temperature EIS measurements; Figure S2: FT-IR spectrum of the calcined SiO_2_NFs; Figure S3: (a) Surface SEM image of prepared PIL-H_2_SO_4_/SiO_2_NF composite membrane. (b) EDS spectra of (a). (c) EDS of mapping of CK, NK, OK, SiK, and SK; Figure S4: (a) Cross-sectional SEM image of the prepared PIL-H_2_SO_4_/SiO_2_NF composite membrane. (b) EDS spectra of (a). (c) EDS of mapping of CK, NK, OK, SiK, and SK; Figure S5: FT-IR spectra of the monomer and the prepared PIL membranes; Table S1: Observed FTIR peaks shown in Figure 3d and Figure S5 and their assignment of monomer, PIL membrane, PIL-H_2_SO_4_ membrane, and PIL-H_2_SO_4_/SiO_2_NF composite membrane; Figure S6: Typical Nyquist plots of the prepared PIL-H_2_SO_4_/SiO_2_NF composite membrane obtained from high-temperature EIS measurements; Table S2: The parameters obtained from the activation energy calculations using Equation (2).
