# Supplementary material for: Poly(Ionic Liquid)-Based Composite Electrolyte Membranes: Additive Effect of Silica Nanofibers on Their Properties
Source: Membranes (Basel). 2025 Aug 27;15(9):254. doi: 10.3390/membranes15090254 (PMC12471868; doi:10.3390/membranes15090254)
Supplement: Supplementary file 1 [file membranes-15-00254-s001.zip › membranes-3821121-supplementary.pdf]

Supplementary Material: Poly(Ionic Liquid)-Based Composite Electrolyte Membranes: Additive Effect of Silica Nanofibers on Their Properties

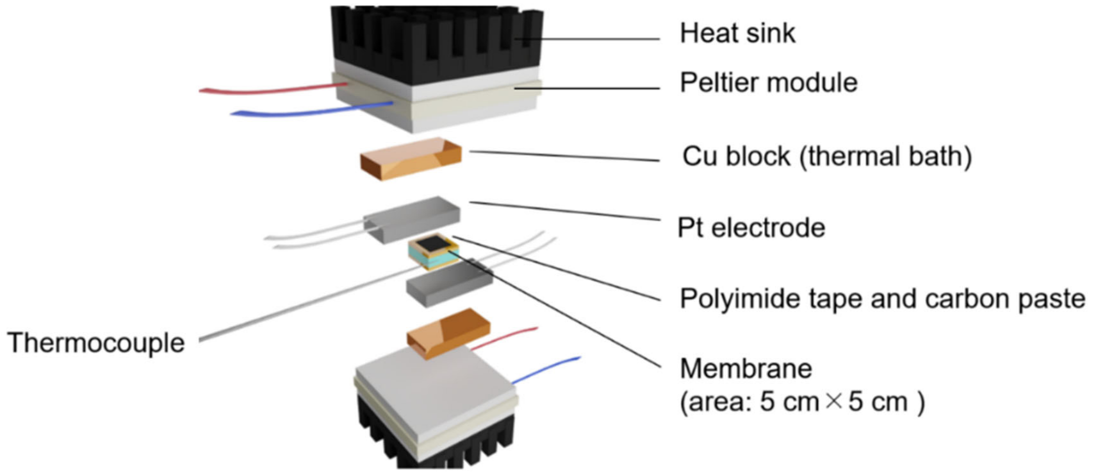

Figure S1. Schematic of the measuring cell for high-temperature EIS measurements.

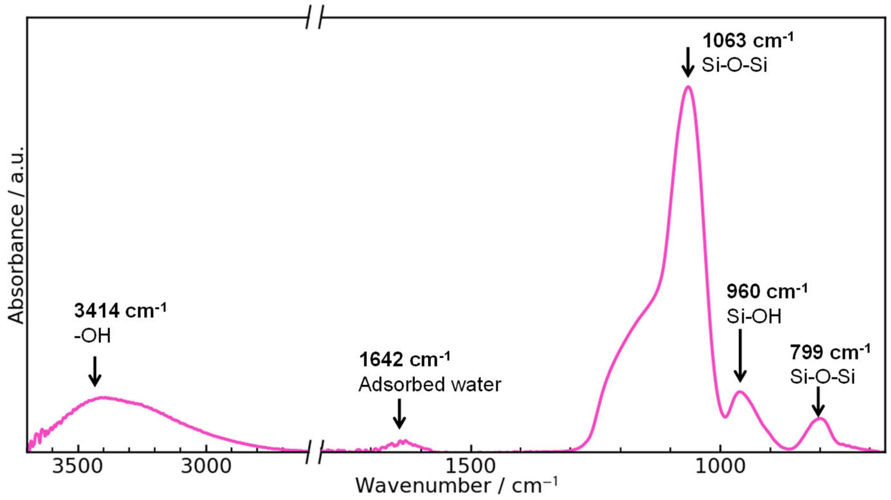

Figure S2. FT-IR spectrum of the calcined SiO_2NFs.

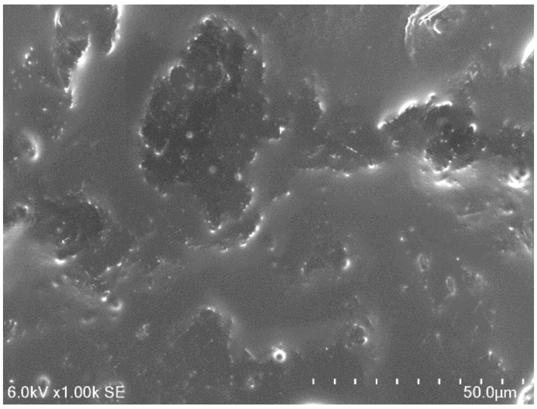

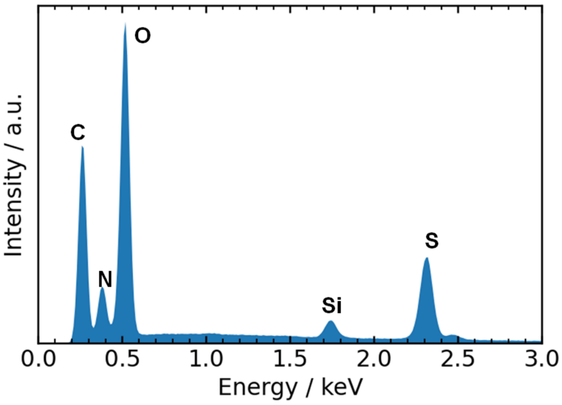

(a) **(b)**

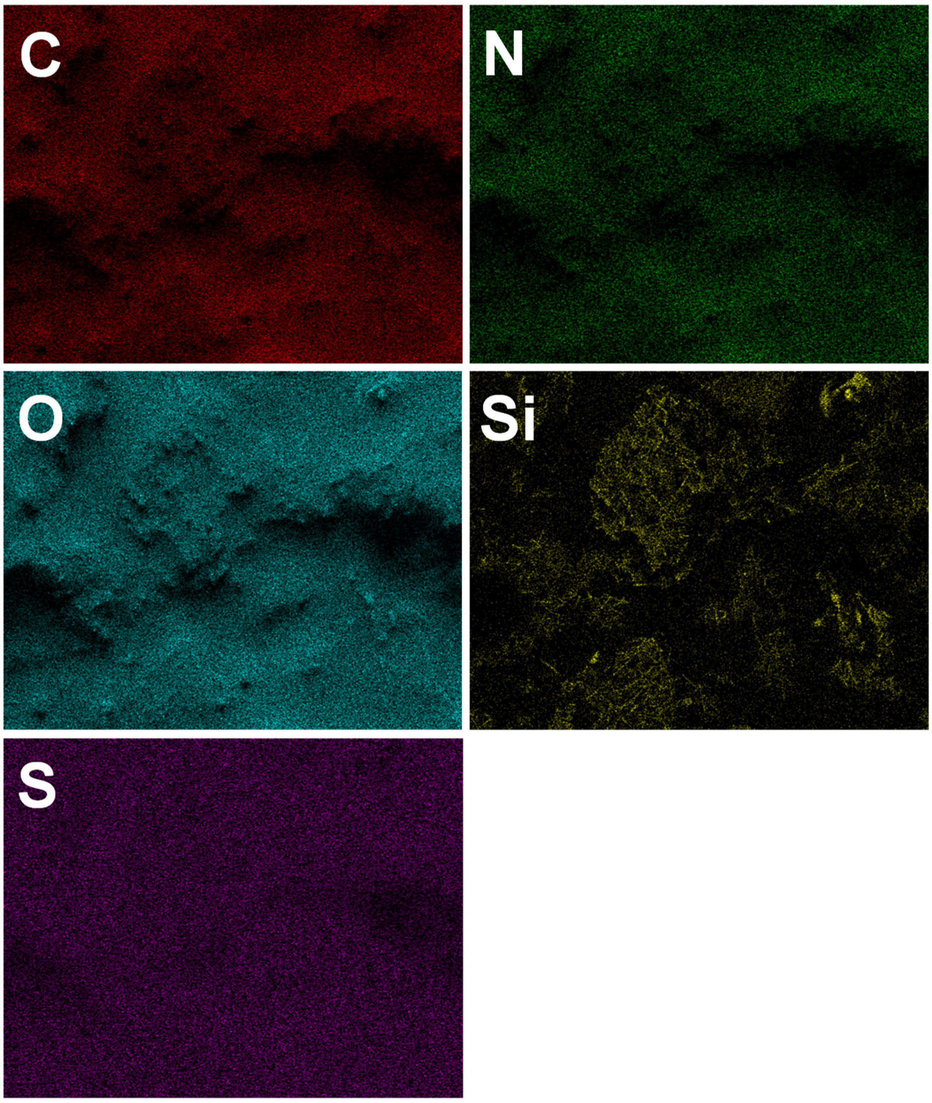

(c)

Figure S3. (a) Surface SEM image of prepared PIL-H_2SO_4/SiO_2NF composite membrane. (b) EDS spectra of (a). (c) EDS of mapping of CK, NK, OK, SiK, and SK.

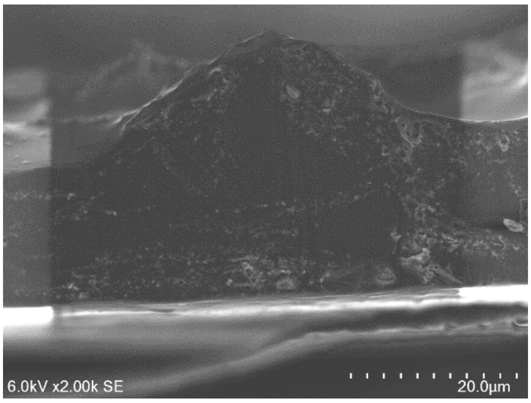

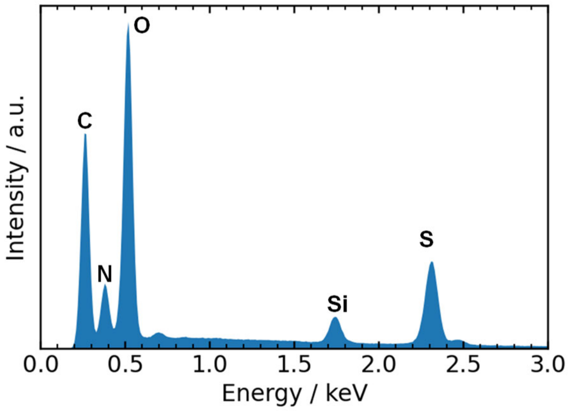

(a) **(b)**

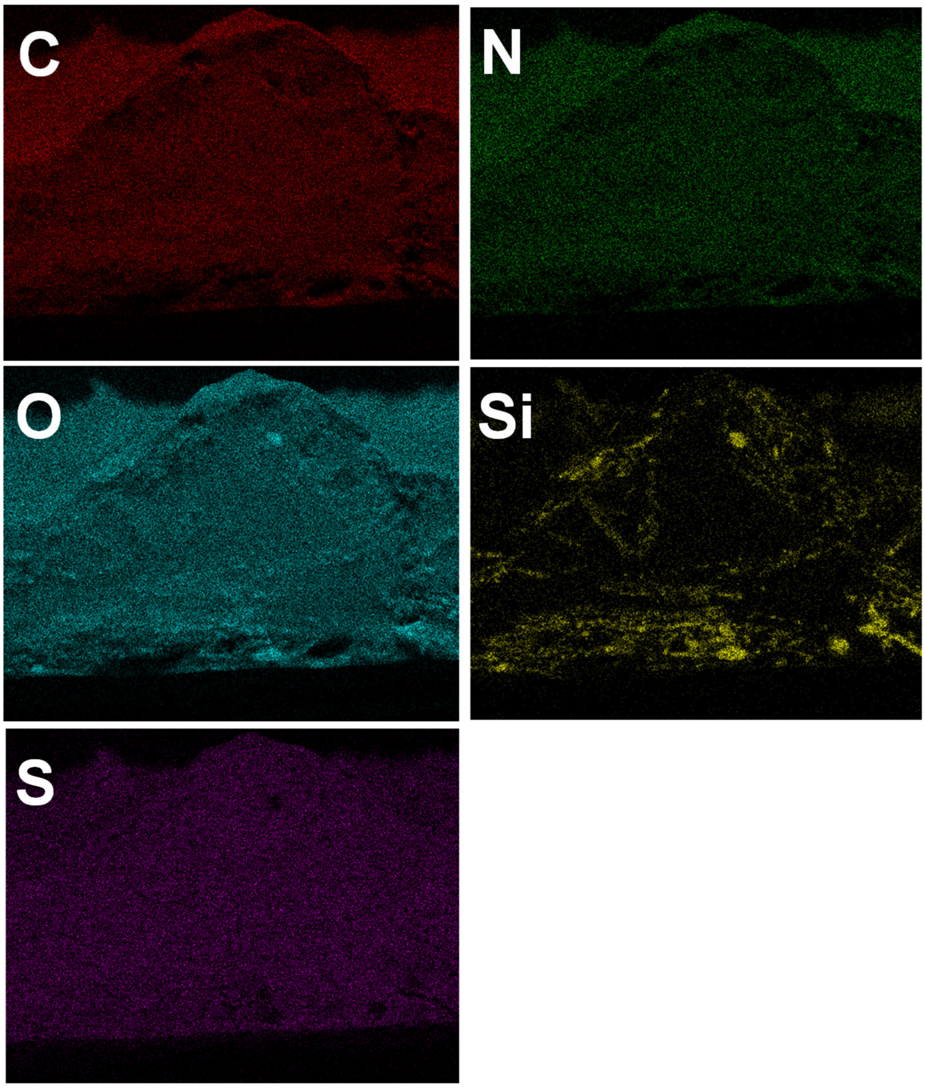

(c)

Figure S4. (a) Cross-sectional SEM image of the prepared PIL-H_2SO_4/SiO_2NF composite membrane. (b) EDS spectra of (a). (c) EDS of mapping of CK, NK, OK, SiK, and SK.

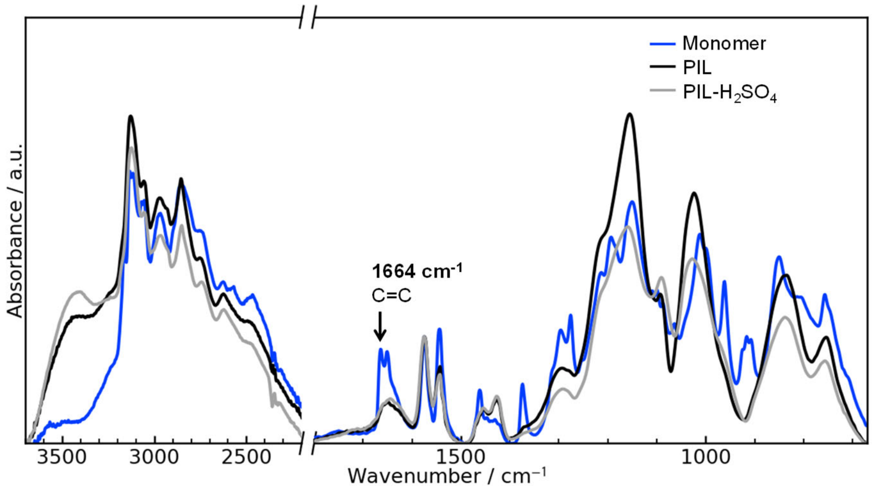

Figure S5. FT-IR spectra of the monomer and the prepared PIL membranes.

Table S1. Observed FTIR peaks shown in **Figures 3d and S5** and their assignment of monomer, PIL membrane, PIL-H_2SO_4 membrane, and PIL-H_2SO_4/SiO_2NF composite membrane. (oop = out of plane, ip = in plane, bend = bending, str = stretching, sym = symmetric, asym = asymmetric)

Observed peak	Peak in references	Assignment	Refs
725-680	702 / 741	Ring oop asym, ip bend	1
757	752-762	Ring HCCH sym bend	1, 2
814-777	762/ 789/790	Ring HCCH asym bend	1, 2, 3
(849)	843/845/846	Ring NC(H)N (CCH) bend	1, 2, 3
	852	S-O str	4
916 (triplet)	930	HSO_4^- ,SO_4^{2-}	5
961	968/970	SO str / S-OH str	5, 6
1000	1004	S-OH bend	6, 7
1012	1010	S=O str	4
1109-1027	1026/1038/1040	SO_3 str, SO str, SO str	1, 3, 8
	1040/1044	CH_2N str	2, 6
	1040/1088	Ring sym/asym str, Ring ip sym bend	1, 3
1150	1129	Ip sym SO_2 bend	8
	1129/1132/1135	SO_2 sym str	1, 3, 8
	1169	Ring ip asym str	1

	1157/1169/1170	(N)CH_2 str	1, 2, 3, 6
	1182/1184	Ring sym str	1, 3
1193	1184	CH_2(N)	3
	1185	H-bonded (OH*O=S), (S=O*) and (S-OH*) str	5
1261-1213	1227	N-C str	2
	1240/1265	Ring ip asym str	2, 6
	1329	SO_2 op asym str	8
1350-1312	1332	CH_2(N) str	2, 3
	1332/1349	Ring ip sym str	3, 3
	1346/1348/1349	SO_2 ip asym str	1, 3, 8
1374	1370	S=O str for H_2SO_4	5
	1410	SO_2 asym str	8
1462-1405	1432/1433	CH_3(N)HCH sym bend	1, 2
	1432/1472	Ring ip asym str	3
	1433	CH_3(N)CN str	1
1544	1544	C=C in graphene	9
	1570/1575	CH_2(N) asym str	2, 3, 6
1576-1576	1574/1579	CH_2(N) CN str	1, 10
	1574	Ring ip asym/sym str	1
1691-1600	1630-1643	deformation(OH) in physically adsorbed water	11, 12, 13, 14
1651	1646	Ring C=C str, N=C-N str	6
1664	1683	C=C in stylene	15
2860	2850	S=O(overtone) asym str of -SO_2OH	11
2967	2990	CH_3(N)HCH asym str	2
2967	3000	OH of -SOH str	5
3069	3070	C_2H str	8
3116	3109	HCCH asym str	8
3134	3126	Ring NC(H)NCH str	1
3162	3149	HCCH sym str	8
3162	3165	Ring ip sym str	1
3162	3165	Ring HCCH asym str	1
3400	3370-3500	OH str of residual water H_3O^+ / N-H str	6, 7, 11, 14, 16

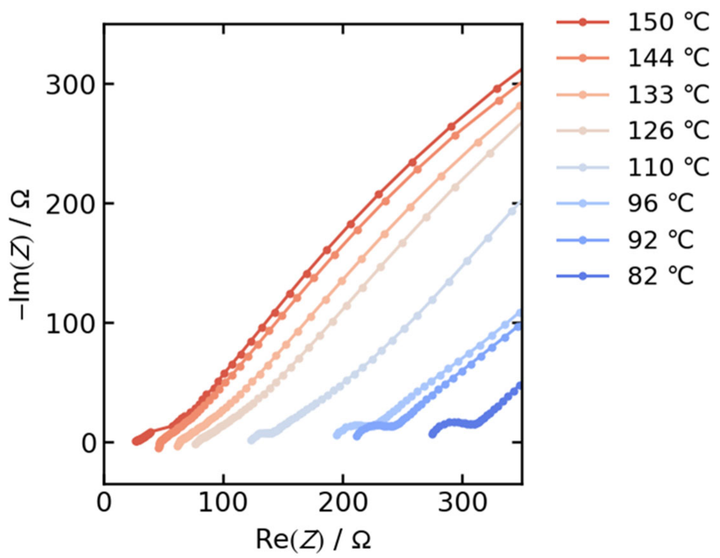

Figure S6. Typical Nyquist plots of the prepared PIL-H_2SO_4/SiO_2NF composite membrane obtained from high-temperature EIS measurements.

Table S2. The parameters obtained from the activation energy calculations using Equation 2.

Membrane	σ_0 [S/cm]	E_a [kJ/mol]	R^2 [-]
PIL	38267	65	0.99
PIL-H_2SO_4	167	44	0.97
PIL-H_2SO_4/SiO_2NF	1442	47	0.99

R^2: coefficient of determination.

References

1. Kiefer, J.; Fries, J.; Leipertz, A. Experimental Vibrational Study of Imidazolium-Based Ionic Liquids: Raman and Infrared Spectra of 1-Ethyl-3-Methylimidazolium Bis(Trifluoromethylsulfonyl)Imide and 1-Ethyl-3-Methylimidazolium Ethylsulfate. *Appl. Spectrosc.* **2007**, *61*(12), 1306–1311. DOI: 10.1366/000370207783292000
2. Moumene, T.; Belarbi, E. H.; Haddad, B.; Villemin, D.; Abbas, O.; Khelifa, B.; Bresson, S. Vibrational Spectroscopic Study of Ionic Liquids: Comparison between Monocationic and Dicationic Imidazolium Ionic Liquids. *J. Mol. Struct.* **2014**, *1065–1066*, 86–92. DOI: 10.1016/j.molstruc.2014.02.034
3. Noack, K.; Schulz, P. S.; Paape, N.; Kiefer, J.; Wasserscheid, P.; Leipertz, A. The Role of the C2 Position in Interionic Interactions of Imidazolium Based Ionic Liquids: A Vibrational and NMR Spectroscopic Study. *Phys. Chem. Chem. Phys.* **2010**, *12*(42), 14153–14161. DOI: 10.1039/c0cp00486c
4. Luo, J.; Yang, Q.; Tan, S.; Wang, C.; Wu, Y. Anisotropic Polymer Membranes Retaining Nanolayered Hydrogen Sulfate Anions for Enhanced Anhydrous Proton Conduction. *J. Memb. Sci.* **2022**, *662*, 120975. DOI: 10.1016/j.memsci.2022.120975
5. Buzzoni, R.; Bordiga, S.; Ricchiardi, G.; Spoto, G.; Zecchina, A. Interaction of H_2O, CH_3OH, $(CH_3)_2O$, CH_3CN, and Pyridine with the Superacid Perfluorosulfonic Membrane Nafion: An IR and Raman Study. *J. Phys. Chem.* **1995**, *99*(31), 11937–11951. DOI: 10.1021/j100031a023
6. Chaker, Y.; Debdab, M.; Belarbi, E. H.; Ilikti, H.; Haddad, B.; Moumene, T.; Wadouachi, A.; Van Nhien, A. N.; Abassi, H. B.; Abbas, O.; Bresson, S. The Influence of Chloride and Hydrogen Sulfate Anions in

Two Polymerised Ionic Liquids Based on the Poly(1-(Hydroxyethyl)-3-Vinylimidazolium Cation, Synthesis, Thermal and Vibrational Studies. *Eur. Polym. J.* **2018**, *108*, 138–149. DOI: 10.1016/j.eurpolymj.2018.08.032
7. Kiefer, J.; Pye, C. C. Structure of the Room-Temperature Ionic Liquid 1-Hexyl-3-Methylimidazolium Hydrogen Sulfate: Conformational Isomerism. *J. Phys. Chem. A* **2010**, *114*(24), 6713–6720. DOI: 10.1021/jp1031527
8. Yaghini, N.; Pitawala, J.; Matic, A.; Martinelli, A. Effect of Water on the Local Structure and Phase Behavior of Imidazolium-Based Protic Ionic Liquids. *J. Phys. Chem. B* **2015**, *119*(4), 1611–1622. DOI: 10.1021/jp510691e
9. Surekha, G.; Venkata Krishnaiah, K.; Ravi, N.; Padma Suvarna, R. FTIR, Raman and XRD Analysis of Graphene Oxide Films Prepared by Modified Hummers Method. *J. Phys. Conf. Ser.* **2020**, *1495*(1), 012012. DOI: 10.1088/1742-6596/1495/1/012012
10. Gu, J.; Luo, J.; Yang, J.; Tan, S.; Wang, C.; Wu, Y. Anhydrous Proton Conduction in Protic Ionic Liquid Crystals Formed by 1-Alkyl-3H-Imidazolium Hydrogen Sulfates. *Ionics* **2022**, *28*(5), 2293–2300. DOI: 10.1007/s11581-022-04481-y
11. Singhal, N.; Datta, A. Reversible Tuning of Chemical Structure of Nafion Cast Film by Heat and Acid Treatment. *J. Phys. Chem. B* **2015**, *119*(6), 2395–2403. DOI: 10.1021/jp506911w
12. Brusatin, G.; Guglielmi, M.; Innocenzi, P.; Martucci, A.; Battaglin, G.; Pelli, S.; Righini, G. Microstructural and Optical Properties of Sol-Gel Silica-Titania Waveguides. *J. Non-Cryst. Solids* **1997**, *220*(2–3), 202–209. DOI: 10.1016/S0022-3093(97)00263-9
13. Rajput, D.; Costa, L.; Terekhov, A.; Lansford, K.; Hofmeister, W. Silica Coating of Polymer Nanowires Produced via Nanoimprint Lithography from Femtosecond Laser Machined Templates. *Nanotechnology* **2012**, *23*(10). DOI: 10.1088/0957-4484/23/10/105304
14. Laporta, M.; Pegoraro, M.; Zanderighi, L. Perfluorosulfonated Membrane (Nafion): FT-IR Study of the State of Water with Increasing Humidity. *Phys. Chem. Chem. Phys.* **1999**, *1*(19), 4619–4628. DOI: 10.1039/A904460D
15. Hermán, V.; Takacs, H.; Duclairoir, F.; Renault, O.; Tortai, J. H.; Viala, B. Core Double–Shell Cobalt/Graphene/Polystyrene Magnetic Nanocomposites Synthesized by in Situ Sonochemical Polymerization. *RSC Adv.* **2015**, *5*(63), 51371–51381. DOI: 10.1039/C5RA06847A
16. Scatena, L. F.; Brown, M. G.; Richmond, G. L. Water at Hydrophobic Surfaces: Weak Hydrogen Bonding and Strong Orientation Effects. *Science* **2001**, 292 (5518), 908–912. DOI: 10.1126/science.1059514
